# Gene Therapy Used in Cancer Treatment

**DOI:** 10.3390/biomedicines2020149

**Published:** 2014-04-08

**Authors:** Thomas Wirth, Seppo Ylä-Herttuala

**Affiliations:** 1 A.I Virtanen Institute, Department of Biotechnology and Molecular Medicine, University of Eastern Finland; Neulaniementie 2, 70211 Kuopio, Finland; E-Mail: seppo.ylaherttuala@uef.fi; 2 Research Unit and Gene Therapy Unit, Kuopio University Hospital, 70210 Kuopio, Finland

**Keywords:** cancer, glioma, gene therapy, gene transfer, viral vectors, non-viral vectors, safety, clinical trials

## Abstract

Cancer has been, from the beginning, a target of intense research for gene therapy approaches. Currently, more than 60% of all on-going clinical gene therapy trials worldwide are targeting cancer. Indeed, there is a clear unmet medical need for novel therapies. This is further urged by the fact that current conventional cancer therapies are frequently troubled by their toxicities. Different gene therapy strategies have been employed for cancer, such as pro-drug activating suicide gene therapy, anti-angiogenic gene therapy, oncolytic virotherapy, gene therapy-based immune modulation, correction/compensation of gene defects, genetic manipulation of apoptotic and tumor invasion pathways, antisense, and RNAi strategies. Cancer types, which have been targeted with gene therapy, include brain, lung, breast, pancreatic, liver, colorectal, prostate, bladder, head and neck, skin, ovarian, and renal cancer. Currently, two cancer gene therapy products have received market approval, both of which are in China. In addition, the stimulation of the host’s immune system, using gene therapeutic approaches, has gained vast interest. The intention of this review is to point out the most commonly viral and non-viral vectors and methods used in cancer gene therapy, as well as highlight some key results achieved in clinical trials.

## 1. Introduction

Cancer is a major global health problem accounting, annually, for more than eight million deaths globally. It is a complex, multifactorial disease involving changes in the genome, which is orchestrated by host and environmental interactions [1]. The hallmarks of cancer are self-sufficiency in growth signals, insensitivity to anti-growth signals, ability for tissue invasion and metastasis, limitless replicative potential, sustained angiogenesis, and evasion of apoptosis [1]. The tumor microenvironment, which is composed of various non-malignant cells expressing various regulatory proteins, as well as the extracellular matrix, plays a pivotal role in the initiation and progression of cancers [2]. Gene therapy aims at delivering genetic material into target cells or tissue and to express it with the intention to gain a therapeutic effect. It has the advantage over conventional therapies due to the fact that it can be administered locally, thereby delivering, locally, a high therapeutic dose without risking systemic adverse effects. Furthermore, since most gene therapies are single time applications, they can be cost effective in the long run.

## 2. Gene Therapy for Cancer: An Overview

Rogers *et al.* was one of the first to demonstrate an initial proof-of-concept of virus mediated gene transfer. What he showed was that foreign genetic material can be transferred to cells of interest by utilizing viruses [3]. Motivated by the results he went even further and tested it in humans. With this experiment, Rogers became the first to perform a human gene therapy trial. In that study, Rogers used a wild-type Shope papilloma virus with the intention to introduce the gene for arginase into two girls suffering from a urea cycle disorder (*i.e.*, hyperargininemias) [4,5]. He hypothesized that the Shope papilloma virus would naturally encode the gene for arginase activity and that this gene could be transferred by introducing the virus to the patients. Unfortunately, the outcome of the trial was negative. There was no change in the arginine levels, nor was there a change in the clinical course of the disease in these patients. Even though Rogers “out of the box” thinking was intriguing, it was doomed to fail as it later turned out that the Shope papilloma virus genome does not encode the arginase gene. 

The US Food and Drug Administration (FDA) approved the first gene therapy protocol, which was carried out in 1989. Therein, tumor infiltrating lymphocytes collected from advanced melanoma patients were *ex vivo* transduced with a marker gene (*i.e.*, not a therapeutic gene), expanded *in vitro*, and re-infused to the patients [6]. The first clinical trial on cancer with an therapeutic intend was started in the following year, wherein patients with advanced melanoma were treated with tumor infiltrating lymphocytes genetically modified *ex vivo* to express tumor necrosis factor [6]. 

Another important milestone in the history of gene therapy was the study conducted by Cline *et al.* Cline treated thalassaemia patients, wherein he extracted bone marrow cells from these patients and transfected *ex vivo* with plasmids containing the human globulin gene. After cells were transfected they were administered back to the patients [7,8]. The reason why this study presents a milestone in the history of gene therapy is not because of the failure of the study itself, but because the study was done without the consent to perform these studies from the University of California, Los Angeles (UCLA) Institutional Review Board. This case demonstrated that knowledge was very limited and that human gene therapy would be technically, as well as ethically much more complex than expected. 

## 3. Gene Transfer Methods and Vectors Used for Gene Therapy

The challenge in gene therapy is to deliver an adequate amount of genetic material into target cells or tissues and to maintain gene expression for a desired period of time. Genetic material can be introduced to their target cells or tissues via different methods of delivery. In principle, we can group them into: (1) physical; (2) viral; (3) non-viral methods; and (4) bacterial or yeast. 

Electroporation, ultrasound, and gene gun deliveries are examples of physical methods that have been used. As the name already implies, with viral vectors a biological (*i.e.*, virus) vector is used as a vehicle to deliver the genetic material into the cells, whereas with non-viral gene transfer methods a synthetic carrier (liposomes or nanoparticles) is used. Different vectors have different properties in relation to their transduction efficiency and their efficacy to express the introduced genes. In addition, they differ in respect of the duration of expression of the transgene, as well as their safety profile. Depending on the requirements, different vectors can be used for different therapeutic purposes. 

Currently, viral vectors are considered as the most effective of all gene delivery methods for *in vivo* gene transfer. Ideally, the gene transfer vector should be able to target a specific tissue with high transduction efficiency and sustain a stable, regulated gene expression without any side effects or immunogenic responses. Unfortunately, none of the currently used gene delivery vectors fulfil all these criteria. Local injection of a vector typically results in a limited, but accurate effect area. On the contrary, systemic administration of a vector can result in a system wide expression. Consequently, vectors and their administration methods have been modified in order to achieve targeted delivery, as well as to increase transduction efficiency [9]. Most viral vectors have, however, already natural tropism to certain cell types or tissues, which can be utilized for therapeutic approaches [10]. 

### 3.1. Viral Vectors

The most commonly used viral vectors used for gene transfer are adenoviruses, lenti- and retroviruses (including the human immunodeficiency virus (HIV)), vaccinia viruses, adeno associated viruses (AAV), and baculoviruses. These vectors differ from each other regarding their cell tropisms, expression profiles, transgene capacities, immunogenicity, as well as different duration of transgene expression. 

In addition to their origin, viral vectors can be divided into integrating and non-integrating vectors. Adenoviruses and baculoviruses are examples of non-integrating vectors. They lack the ability to integrate their genome (and, hence, with it also the transgene) into the host genome. Lenti- and retroviruses, as well as AAVs, on the contrary, are examples of vectors that do integrate into the host genome. While the expression of the transgene is transient in case of non-integrating viral vectors (diminishing in a few weeks), integrating vectors commonly results in long-term expression (months, up to years). This integration of the transgene into the host genome has raised concerns about the safety of these vectors. This is due to the fact that integration has been observed with retroviral vectors to occur occasionally in actively expressed sites (*i.e.*, insertional mutagenesis) [11,12,13]. 

Genetic material can be delivered also by *ex vivo* gene transfer approach. Therein, the genetic material is introduced to the cell outside the patient (*i.e.*, *ex vivo*), into previously isolated autologous cells, which then are re-introduced back to the patient.

Currently, adenoviruses are the most dominant gene delivery vectors used in gene therapy. More than 50 different serotypes have been identified for adenoviruses, which can be divided further into six subgroups (A–F) [14]. Of those, the serotypes 2 and 5 are the most commonly used ones in gene therapy. A limiting factor with adenoviruses is the fact that detectable levels of pre-existing antibodies can be found in 97% of individuals, which potentially may influence transduction efficiency and therapeutic outcome. 

### 3.2. Non-Viral Vectors

Viral vectors have been shown to be efficient gene transfer tools. Nevertheless, drawbacks such as rapid clearance of viral vectors from the bloodstream (when injected systemically), their immunogenic and inflammatory potential, has urged the development of new synthetic gene delivery vectors. In fact, non-viral gene delivery systems are a topic that is currently being studied extensively as alternatives for viral delivery systems. The simplest form of a non-viral system is naked plasmid DNA. The advantage of naked plasmid is that it poses the lowest form of toxicity or other unwanted reactions. In addition, it is easy to formulate and inexpensive to produce. However, its disadvantage is the low transfection efficiency compared to viral-mediated gene transfer [15]. As a result, to improve transfection efficiency, cationic polymers, or lipids formulations have been developed to condense plasmid DNA to protect the degradation of DNA and to enhance uptake and transfection of plasmids [15]. The advantage with those formulations is that polymers or lipids can comparatively easily be designed to attain certain properties. For example, non-viral vectors can easily be targeted to a target tissue or cell by coupling of cell- or tissue-specific targeting moieties on the carrier. Furthermore, by determining the size of the micro- or nanoparticle the biodistribution, cellular internalization, and intracellular trafficking of the micro- or nanoparticle can be influenced [16]. Unfortunately, the success of non-viral delivery systems in clinical applications in gene therapy has been limited. Compared to viral vectors, non-viral vectors have not gone through the evolutionary process of time that viruses have, which typically can be seen as low transduction efficiencies *in vivo*. 

The success of the non-viral gene therapy is dependent on the various extra- and intracellular barriers that affect the efficacy of all gene delivery systems, including cellular uptake, endosomal escape, nuclear uptake, and gene expression [16,17,18].

## 4. Clinical Efficacy of Gene Therapy

Different gene therapy approaches using different gene transfer vectors have been studied for cancer gene therapy. These include induction of apoptosis, oncolytic virotherapy, immune modulation, anti-angiogenic gene therapy, correction of gene defects, inhibition of tumor invasion, gene therapy to enhance chemo- and radiotherapy, myeloprotective gene therapy, antisense and RNA interference (RNAi) based strategies, and pro-drug activation/suicide gene therapy. Unfortunately, only few of these strategies have made it actually to the clinic. One commonly used strategy in cancer gene therapy has been the use of a commonly occurring mutation in the p53 protein. In 2003, Lang *et al.* described a phase I clinical trial using an adenoviral vector encoding for the tumor suppressor gene *TP53* to treat patients with recurrent malignant gliomas, wherein 15 patients ought to undergo intratumoral stereotactic injection of the adenoviral vector via an implanted catheter, followed by *en bloc* resection of the tumor and treatment of the post-resection cavity [19]. Due to the design of the study, tumor response could not be assessed, but the study demonstrated minimal toxicity. No systemic viral dissemination was observed and a maximum tolerated dose was not reached in this study. Furthermore, analysis of tumor specimens demonstrated restricted transgene expression close to the injection site. Another study, and similar to the virus used by Lang *et al.* is Gendicine™. Gendicine™ is a replication-incompetent adenovirus encoding for the *TP53* gene (in place of the viral E1 gene) used for the treatment of a variety of cancers. What makes Gendicine™ interesting is the fact that it became the first gene therapy product that has been approved for clinical use [20]. In a phase I clinical trial, with 12 laryngeal cancer patients, Gendicine™ demonstrated therapeutic potential, as none of the patients treated with Gendicine™ had tumor relapse during the five-year follow-up after the treatment [21] Additionally, Gendicine™ demonstrated good safety profile, exemplified by a phase II/III trials with 132 head and neck squamous cell carcinoma patients. Therein, 32% showed fever as the only side-effect of the treatment [22]. When Gendicine™ was used in combination with radiotherapy, 64% of the patients responded with a complete regression and 29% with a partial regression while with radiotherapy alone, 19% showed a complete regression and 60% a partial regression, suggesting synergistic effect of the combination treatment [22]. 

A second gene therapy product that received market approval by the Chinese SFDA is Oncorine™, developed by Chinese Shanghai Sunway Biotech. Oncorine™ is a conditionally replicative adenovirus, which is produced by deleting the adenoviral *E1B 55K* gene. The deletion of this gene prevents the virus to bind and inactivate the wild-type p53 protein, which is an essential self-defence mechanism of the host against virus infection [23]. When E1B 55K activity is removed, the replication in normal cells is blocked, allowing only replication in p53-deficient cells. In malignant cells the viral proliferation leads to oncolysis, used as a cancer therapy to treat solid tumors. Interesting to this product is the fact that ONYX-015 (developed by Onyx Pharmaceutical’s), which is analogous to Oncorine™, never received market approval. Compared to Oncorine™ ONYX-015 was not able to demonstrate therapeutic benefit in clinical settings. For example, in a phase I dose-escalation trial published by Chiocca *et al.*, 24 patients with recurrent malignant glioma were injected with the oncolytic virus in a total of 10 injections into 10 different sites of the cavity of resected tumors [24]. Even though the study demonstrated that ONYX-015 was safe and that none of the patients experienced serious adverse events that could be attributed to the virus, all patients showed tumor progression. One patient with anaplastic astrocytoma had stable disease and two patients who underwent a second resection had lymphocytic and plasmacytoid cell infiltration at the site of injection. 

Oncorine™ and ONYX-015 have together provided a vast amount of safety data for various types of cancer, including glioma, head and neck, pancreatic, and ovarian cancers, demonstrating an acceptable safety profile [20]. Typical complications included fever, injection site pain, nausea, alopecia, leucopenia, and flu-like symptoms [25]. 

In order to improve efficacy of oncolytic viruses, additional therapeutic proteins have been added to the viruses. An example for this is Onco VEXGM-CSF, which is a second-generation oncolytic herpes simplex virus (HSV), additionally coding for the therapeutic protein granulocyte macrophage colony-stimulating factor. A phase I safety study showed that Onco VEXGM-CSF was well tolerated and safe when administered by intratumoral injection in patients with cutaneous or subcutaneous deposits of breast, head and neck and gastrointestinal cancers, and malignant melanoma who had failed prior therapy [26]. In addition, evidence of an antitumor effect was seen in that study, which was further supported by a Phase I/II, in which Onco VEXGM-CSF was given in combination with radiotherapy and cisplatin to patients with untreated stage III/IV squamous cell cancer of the head and neck [27].

### 4.1. Gene Therapeutic Approaches to Stimulate the Immune System

Immunotherapy is a topic that has gained much attention recently. Typically, in immunotherapy the objective is to enhance either the recognition or presentation of tumor-associated antigens (TAA’s). Unfortunately, there are common challenges that have been faced by immunotherapies, including the natural tolerance towards TAAs and the strongly immunosuppressive tumor microenvironment. Particularly, the genetic engineering of T cells has been of intense research [28]. An example for genetic engineering of T cells is the introduction of a T cell receptor (TCR) against a known TAA. An example of such an approach is the clinical report by Morgan *et al.*, wherein they transduced normal peripheral blood lymphocytes (PBLs) using retroviral vectors with an anti-MART1 TCR transgene that was isolated from tumor infiltrating lymphocytes (TILs) of patients with melanoma [29]. Therein, they demonstrated durable engraftment of the T cells in 15 patients at levels exceeding 10% of peripheral blood lymphocytes for at least two months after cell infusion. Furthermore, they observed high sustained levels of circulating, engineered PBLs at one year after infusion in two patients who both demonstrated objective regression of metastatic melanoma lesions. In another clinical trial T cells were transduced with a TCR against the antigen NY-ESO-1, a cancer/testis (CT) antigen expressed in various cancers [30]. In addition, in this trial, an objective clinical response in patients was observed, providing evidence that introduction of a TCR targeting a TAA represents a feasible option for the treatment of cancer.

Similarly to introducing a TCR an artificial T cell receptor (typically referred to as a chimeric antigen receptor; CAR) can be introduced to T cells. Utilizing CAR to target T cells to cancer cells has resulted in impressive response rates in the clinic against haematological malignancies [31]. An example is the study performed by Kochenderfer *et al.*, who assessed in a clinical phase I trial the potential and safety of adoptive transfer of genetically modified T cells expressing CAR against CD19 [32].

Another way to boost an anti-tumoral immune response was evaluated by Herman *et al*. in a randomized phase III clinical trial among patients with locally advanced pancreatic cancer [33]. A second generation replication-deficient adenovirus of the serotype 5 containing the TNF-α cDNA under the early growth response protein 1 (Egr-1) promoter was assessed for this purpose. The Egr-1 is a promoter, which is induced by ionizing radiation, thus restricting the expression of the transgene to the radiation field. In that study, 304 patients were randomly assigned 2:1 to standard of care plus gene therapy (*i.e.*, adenovirus encoding for TNF-α) *versus* standard of care alone. The results revealed that even though standard of care plus gene therapy was safe it did not result in a survival benefit in patients with locally advanced pancreatic cancer [33]. A more promising result, in contrast, was presented in a study by Malmström *et al.*, wherein they studied the immunostimulating effects of gene therapy with adenoviral vectors expressing CD40 ligand [34]. CD40L belongs to the TNF gene superfamily and is known to be a potent immune stimulator of T helper 1 cells. This study recruited eight patients with invasive bladder cancer for a phase I/IIa trial evaluating the safety, efficacy of gene transfer, immune effects, and antitumor responses [34]. The results demonstrated that the presence of IFN-γ was increased in the biopsies of tumors, whereas levels of circulating T regulatory cells were reduced. Further histologic evaluation indicated that adenoviral CD40L gene therapy reduced the load of malignant cells in the bladder. 

In another study performed by Chiocca *et al.*, 11 patients were injected with different doses of interferon-β-expressing adenoviruses ranging from 2 × 10^10^ to 2 × 10^11^ viral particles stereotactically into the tumor [35]. This was followed by surgical removal of the tumor four to eight days later with additional injections of the adenovirus into the tumor bed. Unfortunately, all patients had disease progression and/or recurrence within four months of the treatment. The median time to tumor progression was 9.3 weeks and the median overall survival was 17.9 weeks.

In addition to the above mentioned strategies, the utilisation of a pro-drug activating suicide gene therapy is an approach that has been extensively explored pre-clinically and in the clinic for cancer therapy, which will be discussed in more detail below.

### 4.2. Pro-Drug Activating Suicide Gene Therapy

The principle of pro drug activating suicide gene therapy is to introduce a transgene encoding for an enzyme that is either absent in mammalian cells or present in a very inactive form, into the tumor. The enzyme produced by the transduced cells will convert the subsequently administered inactive pro drug into its active form, evoking the death of cells expressing the therapeutic gene. Therein, the bystander effect (a phenomenon wherein also the neighboring non-transduced cells are killed) is fundamental for the therapeutic success [36]. In this concept, brain tumors bear several features that make them particularly amenable to pro-drug activating gene therapy. First of all, brain tumors are typically single, localized lesions of rapidly dividing cells in a background of non-dividing cells. Furthermore, recurrence typically happens in the close vicinity of the original lesion. Unfortunately, the first results were not very promising. Transduction efficiency was a major problem resulting in a poor therapeutic efficacy. The use of retroviral vectors in those early studies was most likely a major reason for poor transduction efficiency. In comparison to retroviral vectors, adenoviral vectors have shown to have much higher transduction efficacy as well as transgene expression [37]. One of the reasons is that in comparison to retroviruses, adenoviruses transduce both dividing and quiescent cells. This feature may provide an important advantage, as not all cancer cells proliferate within the tumor at a given time point. In 1996, Eck *et al.* published the first phase I clinical trial, where the pro-drug activating enzyme Herpes simplex virus—thymidine kinase (HSV-tk) packed into an adenovirus was used with the intention to treat patients with recurrent gliomas [38]. The first completed trial using adenovirus HSV-tk in patients with malignant glioma, however, was published by Sandmair *et al.* in 2000 [37]. In that study, Sandmair *et al.* compared the efficacy of both the retrovirus-packaging cells for HSV-tk and the adenovirus mediated HSV-tk gene therapy for the treatment of primary or recurrent gliomas. Twenty-one patients were enrolled in that study. The mean survival time in the adenovirus HSV-tk group was 15 months and significantly longer when compared to a 7.4 months survival time in the retrovirus-packaging cell group. The control group, which received adenovirus LacZ had a mean survival time of 8.3 months. Although the retrovirus-packaging cell approaches were found safe, no efficacy was observed. The low gene transfer efficacy with retrovirus and the lack of the treatment response indicated that retroviral HSV-tk gene therapy may not be efficient enough in human clinical settings. The lack of efficacy was further confirmed in the first randomized, open-label, parallel group phase III clinical trial of 248 patients, where HSV-tk was produced by retroviral producing cells. The study did not show any improvement of survival [39]. The clinical efficacy of HSV-tk gene therapy was first demonstrated in two separate phase II clinical trials; a phase IIa trial and a randomized and controlled phase IIb trial [37,40]. Therein, 17 patients with operable or recurrent malignant gliomas receiving HSV-tk gene therapy out of 36 patients implicated a survival advantage over control patients, who did not receive HSV-tk gene therapy [40]. The mean survival of the patients treated with HSV-tk gene therapy was significantly longer (*p* < 0.0095) when compared to the standard care group or a historical control group (*p* < 0.0017). This study was also historically the first randomized, controlled trial with an adenoviral vector using the HSV-tk pro-drug activating suicide gene, where survival benefit could be demonstrated. Encouraged by these results, a multicenter, standard care controlled, randomized clinical phase III trial was commenced. Therein, 250 patients were recruited and randomly allocated, whereof 124 were allocated to the experimental group and 126 to the standard care group. The median time to death or re-intervention was longer in the experimental group (308 days) than in the control group (268 days). Interestingly, in a subgroup of patients with non-methylated status of the DNA repair gene *MGMT* (O6-alkylguanine DNA alkyltransferase), the hazard ratio (HR) was 1.72 (*p* = 0·008). However, no statistical difference in the overall survival between the groups was observed [41]. Although the study did not demonstrate improvement of overall survival, the findings suggested that the use of HSV-tk gene therapy after tumor resection can increase time to death or re-intervention in patients with newly diagnosed supratentorial glioblastoma multiforme. Furthermore, this study demonstrates that locally delivered gene therapy for glioblastoma should be further developed, especially for patients who are unlikely to respond to standard chemotherapy. This study is, thus far, the only adenoviral vector study that has completed a phase III clinical trial, which is based on the suicide gene therapy with HSV-tk. 

## 5. Safety of Gene Therapy

Despite the tragic case of Jesse Gelsinger, who died as a result of gene therapy using adenoviral vectors, the safety data collected from different human gene therapy trials have been uniformly satisfactory. However, it should be pointed out that viral vectors used in gene therapy are typically human pathogens, and hence, pre-existing antibodies against the viral vector may be present, which might result in an unwanted immune response. For example, an injection of adenoviral vectors will result in an initial non-specific immune response in the host, *i.e.*, release of a variety of cytokines followed by a specific antibody and cell-mediated immune response directed against transduced cells. However, the response towards adenoviruses is serotype dependent. For example, a study by Thoma *et al.* demonstrated that the immediate cytokine response of macrophages following adenovirus stimulation differs between adenovirus serotypes, hence, is serotype-specific. Particularly, in a long-term study, wherein either adenovirus of the serotype 11 (Ad11) or 5 (Ad5) was administered intra peritoneally, Ad11 caused no/mild and Ad5 moderate/severe toxicity [42].

Generally, there is still not much long-term safety data using viral vectors in humans. Nevertheless, several meta-analysis already exist for adenoviruses demonstrating an adequate safety profile in humans [41,43]. The tolerability towards adenoviral vectors has been acceptable and the side effects have mostly been mild without any serious adverse events related to gene therapy. 

Different means with the intention of improving the safety of gene therapy have been implemented. One approach is to develop targeting strategies in order to enhance the delivery of gene transfer vectors, and hence, to improve the duration and efficacy of gene expression. Generally, one of the major shortcomings with gene therapy is their lack of specificity to their target cells and their low transduction efficiency. Improving specificity and/or transduction efficacy ultimately would result also in a better safety profile. Consequently, the improvement of transduction efficacy of gene transfer vectors has come along with the development of vector technologies, including re-engineering of viral vectors using epitope insertion, chemical modification, and molecular evolution [44]. An example for this was demonstrated in a phase I clinical trial by Kim *et al.*, where they modified the RGD fiber knob on adenoviruses, thereby enhancing viral infectivity of cancer cells [45].

The role of innate immunity, as well as the activation of T and B cells in response to the vector and its transgene product is a topic of intense research. Particularly, the possible effects of gene transfer vectors and/or their expressed proteins on local lymph nodes are topics that require further evaluation. The pre-existence of neutralizing antibodies (e.g., against several adenovirus serotypes or AAVs) has been acknowledged already for quite some time and it is known that these pre-existing neutralizing antibodies can substantially diminish transduction efficiency [46]. 

In order to improve specificity, as well as transduction efficiency, viral surface proteins have been modified, removed or replaced. For example, lentiviral vectors have been generated, wherein a cell type specific ligand or antibody has been fused to the viral envelope (*i.e.*, pseudotyping) [47]. The downside of this has been that different modifications resulted in low vector titers during lentivirus production [13]. Furthermore, it has been shown that targeting may also potentially compromise the entry of the vector into the cell [13,47]. On the contrary to targeting viral vectors to specific cells, pseudotyping can also be used to broaden tropism of the viral vector to other cells. For example, retroviruses and lentiviruses are frequently pseudotyped with the Vesicular Stomatitis virus G-protein (VSV-G) to widen their tropism and to increase their yield in production [48]. 

Another approach to increase specificity of viral vectors to their target cells is the use of tissue-specific or conditional promoters. An example for conditional dependent gene expression is the use of hypoxia-specific regulatory systems, where gene expression is aimed to be induced and restricted to ischemic tissues [49]. Commonly, these hypoxia-specific regulatory systems have been applied to various ischemic disease models, including ischemic myocardium, stroke, and injured spinal cord, but could also be used in cancer gene therapy [50]. Gene expression can also be regulated based on a genotypic feature (e.g., a mutated *TP53* gene in cancer cells), which has been discussed already above in case of Oncorine™.

The risk of insertional mutagenesis with integrating vectors is a safety risk. Retroviruses, lentiviruses and AAVs are examples of viruses that integrate their genome into their host chromosomes. By doing so, there is a chance that these vectors may integrate into gene regulatory areas or into transcriptionally active areas, respectively, which potentially can adversely result in insertional mutagenesis and oncogenesis. Several approaches have been developed to circumvent these problems. Therefore, targeted integration of transgenes to predetermined genomic sites has been one of the most important topics in current vector development. One of the most efficient methods to achieve targeted integration into human cells is based on DNA double-strand break-enhanced homologous recombination [51]. In addition, lentivirus/transposon hybrids have been developed in order to reduce the risk of insertional mutagenesis [52]. For example, the Sleeping Beauty transposon system is an attractive approach allowing stable integration of the transgene through transposition into the target cell genome [53,54]. The advantage of the Sleeping Beauty transposon system is that it does not exhibit a preference for integration within active genes and the inverted repeats have only very low residual promoter/enhancer activity. The risk of genotoxicity/mutagenesis caused by gene therapy has been one of the main arguments against human gene therapy is. However, the fact, that conventional cancer therapies (*i.e.*, radiation therapy and chemotherapy) also can cause genetic alterations is often disregarded. It is fact that many chemotherapeutic drugs, as well as radiation therapy, may cause genetic alterations and oncogenesis in patients [55,56,57]. 

In addition, by developing the manufacturing of gene transfer vectors (*i.e.*, development of production cell lines, production methods, as well as the purification steps) the safety profile of gene transfer vectors can be improved. For example, gutless adenoviral vectors are vectors, where all other genes but those essential for virus production are removed and replaced with the gene of interest, driven by a suitable promoter. As a result, gutless adenoviruses still exhibit high transduction efficiency and similar tropism to previous vectors, but are less immunogenic than the first generation adenoviral vectors. However, since gutless vectors are devoid of all viral genes, co-infection with a helper adenovirus is required that provides proteins needed for its genome replication, packaging, and capsid formation. As both helper and gutless vectors have the same viral capsid, separation must be addressed before purification, which is laborious and has not been without challenges [58].

## 6. Conclusions

Gene therapy is an intriguing and potential approach to treat various diseases, including cancer. Currently most gene therapy protocols are limited to the local administration of the gene transfer vector, or to *ex vivo* gene transfer approaches. One of the challenges in gene therapy is still the low transduction efficiency and its minimal distribution of the vector within the tissue. However, it should be emphasized that focus should not only be directed towards vector development itself, but also towards the manufacturing of these vectors. The high cost involved in viral vector manufacturing, which is the result of tedious downstream purifications steps, has been challenging. In addition, the concept of using gene therapy as a single agent therapy has not been as successful as being hoped. Consequently, combination therapy with existing conventional modalities or other new therapies should be considered and may offer additional benefit in cancer gene therapy.
